# “Boo-Khar”—India Fever

**Published:** 1889-06

**Authors:** Alice B. Condict

**Affiliations:** Hyderabad, Deccan, India


### *"BOO-KHAR"—INDIA FEVER.*

BY ALICE B. CONDICT, M. D., Hyderabad, Deccan, India.

TO THOSE who are interested in the study of the antique, this old Arabic word, meaning "fever," may have an interest, especially when we find it is still in use by about one-sixth of the world's population, viz.: India, Arabia and Persia.

Long before our now dominant English language was framed or formed, this Arabic word was in use by those fathers of medicine. It comes from a land where centuries glide over the white-robed forms and wise-looking turbaned heads of "The Henckeems" (doctors), without seeming to enlarge or change their modes of thought or treatment.

With them the practice of medicine still savors strongly of necromancy and the supernatural, showing that what we denominate "the newly-discovered mind cure" is older than our own history or language, and has been practised from the earliest recorded times. We will, however, deal only with the more practical side of our "Henckeem" friends' mode of treatment.

In this part of the world, "Boo-khar" is preëminently the most frequent malady we are called to treat; the most productive cause being either exposure to the mid-day sun, or the sudden congestion of the relaxed system by cold.

Sunstroke, as seen in America, is seldom found here; isolated cases always being Europeans who have recently come to the country. The daily papers of New York have startling records to make of sunstroke during a hot season in July and August,—men falling insensible in the street, numbers of horses falling dead in a short time.

The sudden giddiness, the unconscious period, when the poor victim is picked up in the street and carried to the hospital or nearest drug store, is almost an unheard-of circumstance here. Instead of such effects, we find sudden rise of fever that runs a rapid course, with high temperature, that may prove fatal in a few hours, with a history of exposure to mid-day sun. The after-effects, however, are identical with the sunstroke in temperate climates, morbid conditions of the brain being frequently set up. Fever, also, frequently comes from exposure to the occasional penetrating mist of early morning that is dissipated by the rising sun. Very significantly, this mist is also called "Boo-khar," the popular opinion being that there is a strong relation between the two.

The native of India protects himself when he must meet it, as we would to meet a snow-storm in America, bundling himself up head and ears to escape its chill. Although the thermometer does not show a marked reduction of temperature, yet in the relaxed state in which dwellers of "The Tropics" are apt to be, a sudden fall of from five to ten degrees is keenly felt.

Notes gleaned from English officers and surgeons who have lived in India almost a life-time with scarce a severe attack of fever, give us some important precautions, which, if carefully followed out, will insure comparative freedom from fever in India.

Having tested the following rules for some years, we give them here, believing that even in America they may prove useful during the heated term:

First.—Never go into the mid-day sun without a genuine sun-protector hat (heavy felt and pith), and a two-fold covered umbrella. Even with these precautions, avoid walks at mid-day as far as possible.

Second.—Wear a flannel garment that covers the entire abdomen, next the skin, fitting closely. Let it be thin, but all wool. This is termed the "cholera belt," and is a great protection against the dread disease.

Third.—Never allow yourself to sit in a draught, so that you are chilled, or wear clothing wet from perspiration, and thereby get chilled.

These precautions limit the severity and frequency of attacks of fever, even when one must live in a malarious climate.

It is interesting to note that even when the system is more or less under the poisonous influences of malaria, it will successfully throw it off by excretion, so long as the glands and excretory ducts are not clogged by congestion, such as is rapidly set up by cold. If the strain be temporary, there may be no evil results; but when the glands are weakened from inaction, or have become flabby from exposure to long-continued heat, and the congestion lasts some hours, they break down, and our patient has pneumonia, or enlarged spleen, or congestion of the liver,—all or any of which will be accompanied with fever, and frequently ague.

To relieve the intense bone pains of most kinds of fever, our "Henckeem" friends prescribe a species of massage, that is heroic in its quality compared to the delicate manipulation of the skilled masseuse of Europe or America.

Every Arabic or Indian family employ an "Ayah" (nurse), to give this very favorite luxury to the sick or ailing members of the family. She kneads the extremities and body in a thorough manner, pressing deeply with the palm of the hand till every drop of capillary blood is displaced, and, although the uninitiated may at first cry out from her severe treatment, yet the immediate after-affect is so comforting that the patient begs to have the operation repeated over every portion of the body, again and again. Indeed, it is generally continued hour after hour in a variety of modes—percussion, rolling, and beating with the finger-tips being a part of the process.

Even the horse-keepers use this method to keep their horses in a fine condition. Especially after a drive in the mid-day sun, they "mallaise" them well. An especially fine Arab horse will have two men, who give the "mallaise" daily.

At mid-day, a horse-car driver in Bombay was seen lying flat on the ground, in the shade of a tree, while a friend, with his bare feet, walked with heavy measured tread up and down the naked body of the prostrate man.

Curiosity induced us to wait to see the operation, which we were told was to relieve the bone pains of the fever the poor fellow had contracted from the sun. After fifteen minutes, he was up and off on his street-car to the other end of his line, professedly greatly relieved by this heroic remedy.

Of drugs, none seem to be more popular than aconite during the fever; and quinine in some form after. Aconite we know is indigenous to India. Reliable parties, who have traveled through the dense jungles of upper India, tell us of small rivulets whose waters have become impregnated in their flow over the aconite roots with its poisonous qualities; the natives having warned our traveler friends of the danger of drinking the water, by saying, "Meeta zeer haigh" (Sweet poison is there).

The curative qualities of aconite during the congestive stage are due to its physiological action in relaxing the walls of the arterial system, thus, as it were, bleeding the patient into his own veins, giving more room, lessening pressure on the overtaxed internal organs, and thus tiding him over the critical period.

Quinine is also grown in the mountainous districts of India, "Darjeeling Chincona" being much used by "Henckeems," as well as in our own practice. A tonic dose suits the patient well who has been treated by aconite.

Time and space fail me to describe the crude and semi-barbaric modes used by our "Henckeem" friends, from the rude red-hot iron to the expensive and elegant prescription of "ashes of pearls." The last-named remedy is highly valued by the nobility, many of whom deem it a proper remedy for every variety of disease!

The red-hot iron is frequently resorted to when the patient is supposed to be suffering from a visit from the devil!

No doubt, American doctors would sometimes be glad of so simple a solution of difficulties in cases of difficult diagnosis.

HYDERABAD, April 10, 1889.

---

THE American Medical Association holds its Fortieth Annual Meeting in Newport, R. I., June 25th, 26th, 27th and 28th. Coincidentally therewith Newport celebrates its 250th anniversary.

